# News and Coming Events

**Published:** 1908-02-01

**Authors:** 


### NEWS AND COMING EVENTS.

On Wednesday, February 12, at 8 p.m., at the Royal Sanitary Institute, Parkes Museum, Margaret Street, W., a discussion will take place on "Rivers Pollution, with special reference to the Board proposed by the Royal Commission." Sir William Ramsay will open the discussion, and the chair will be taken by Sir Alexander R. Binnie.

The City Board of Aldermen of New York, on January 21, passed an ordinance forbidding women to smoke in public places. Tacit permission has been given for women to smoke cigarettes at several expensive restaurants since New Year's Eve, when one of the leading restaurants suspended its order against the practice.

The High Commissioner for New Zealand desires to direct special attention to the fact that persons suffering from consumption and unable to provide for their maintenance inside a sanatorium, cannot be allowed to land in New Zealand. Indigent sufferers are required to return by the next boat leaving after their arrival, and it is therefore specially important that consumptive patients of that class should be warned not to go to New Zealand.

The Seaforth Sanatorium, built and endowed by Colonel and Mrs. Stewart-Mackenzie, of Seaforth, for the reception of patients suffering from phthisis in its incipient form, was formally opened on January 16 by Mrs. Stewart-Mackenzie. The foundation-stone of the building was laid in November 1906, and, although the institution will not be occupied until May or June, it is now completed and practically ready for the reception of patients.

In consequence of certain reports which appeared in the Press in relation to the Versailles affair, Dr. P. Z. Hebert issued writs against the *Daily News*, the *Daily Mirror*, the *Daily Mail*, the *Evening News*, the *Pall Mall Gazette*, the *Evening Standard*, the *Daily Express*, the *Westminster Gazette*, the *Manchester Courier*, the *Glasgow Evening Times*, and the *Liverpool Echo* for publishing libellous articles referring to Dr. Hebert in connection with a charge at Versailles against the woman Pesnel, *alias* Cesbron. The case has now been settled out of Court by the payment to Dr. Hebert of the sum of £1,000 for damages and costs and the undertaking on the part of the said newspapers to publish an apology of a form agreed upon. Messrs. Roberts Seyd and Co., of Regent Street, represented Dr. Hebert.

At the quarterly meeting of the Board of Delegates of the Hospital Saturday Fund Association, held at the Central Offices, Gray's Inn Road, the secretary (Mr. A. W. Davis) reported that the receipts from the industrial establishments etc., from January 8, 1907, to Saturday last had amounted to £22,804 13s., being a slight increase as compared with the corresponding period of the previous year. The report of the Council, which was adopted, showed that the endowed beds in the Chest Hospitals and sanatoria connected with the Fund had been kept fully occupied, and satisfactory testimony had been received with regard to the benefit derived by the patients sent to these institutions. Since January 8 last 1,574 letters for convalescent homes had been issued, of which number 689 were part-pay letters, the patients paying £953 2s. 10d. toward the cost of maintenance. In the surgical appliance department the amount received from patients as part payment for various appliances, etc., had reached £4,055 5s. 4d. The work in connection with the new dental department had given general satisfaction. The Lord Mayor of London (Sir John Bell) has consented to act as president during his year of office.

Dr. Clement Dukes has been appointed consulting physician to Rugby School.

Lord Glenesk will preside at the 69th annual general meeting of the Newsvendors' Institution, to be held at Memorial Hall, Farringdon Street, in the City of London, at seven o'clock on Wednesday evening, February 19.

Professor William Osler, M.D., F.R.S., Regius Professor of Medicine at Oxford, and Dr. Arthur Latham, physician to St. George's Hospital, have been chosen to fill vacancies on the council of the National Association for the Prevention of Consumption.

The Lettsomian lectures for 1908 on "Tuberculosis of the Kidney" and "Malignant Disease of the Cæcum" will be delivered by Mr. Charles J. Symonds, M.S., F.R.C.S., on February 3 and 17 and on March 2, at 9 p.m., at the Medical Society of London, 11 Chandos Street, Cavendish Square, W.

At a meeting of the governing body of Downing College, Cambridge, held on January 25, Sir Herbert Isambard Owen, M.D., F.R.C.P., senior Deputy-Chancellor of the University of Wales, and Principal, Armstrong College, Newcastle-on-Tyne, was elected as Honorary Fellow of the College.

Another instance of abdominal surgery performed under trying conditions is reported in the current issue of the *New York Medical Record*. A coal trimmer on board the Cunarder *Pannonia* on December 23 manifested symptoms of acute appendicitis, and the ship's surgeon, Dr. J. Fraser Orr, decided that prompt operation was imperative. As there was a heavy sea on, the engines were stopped and the ship allowed to fall into the trough of the waves to lessen the pitching while the surgeon, with the aid of a medical assistant, removed an appendix that was found to be gangrenous. On reaching port the patient seemed in good condition and likely to have an uncomplicated convalescence.

Lord Lister was on January 22 enrolled as honorary burgess of the city of Glasgow. The ceremony took place in St. Andrew's Hall, in the presence of a large and representative gathering of citizens. Owing to the condition of his health, Lord Lister was unable to attend personally. The Lord Provost, in making the presentation, recalled Lord Lister's connection with the city while professor of surgery at the University and visiting surgeon at the Royal Infirmary. Professor Sir Hector Cameron, one of Lord Lister's former students and his lifelong friend, accepted on Lord Lister's behalf the casket containing the burgess ticket, and read from him a letter recalling his connection with the University and the city.

The Tobacco Dealers' License Reform Association suggests that an increase in the price of tobacconists' licenses will help to solve the problem of how to lessen juvenile smoking. They maintain that children do not obtain cigarettes from responsible tobacconists, but from the little "hucksters," who, owing to the anomalies of the licensing laws, can obtain a dealers' license for 5s. 3d., and by this trifling outlay are placed on the same footing as the largest store. An increase in the tobacco dealer's license would have a distinctly repressive effect upon juvenile smoking, and respectable tobacconists would welcome the change. Legitimate tobacco dealers, according to the Association want two things—a higher license and the imposition of proof of respectability as a qualification. A meeting of tobacconists who are in earnest in desiring this reform is to be held this week with a view to memorialising the Chancellor of the Exchequer in the matter.

